# Characterization of De Novo Synthesized GPCRs Supported in Nanolipoprotein Discs

**DOI:** 10.1371/journal.pone.0044911

**Published:** 2012-09-28

**Authors:** Tingjuan Gao, Jitka Petrlova, Wei He, Thomas Huser, Wieslaw Kudlick, John Voss, Matthew A. Coleman

**Affiliations:** 1 NSF Center for Biophotonics Science and Technology, University of California Davis Medical Center, Sacramento, California, United States of America; 2 Department of Biochemistry and Molecular Medicine, University of California Davis Medical Center, Sacramento, California, United States of America; 3 Department of Radiation Oncology, University of California Davis Medical Center, Sacramento, California, United States of America; 4 Life Technologies, Carlsbad, California, United States of America; 5 Lawrence Livermore National Laboratory, Livermore, California, United States of America; University of Oldenburg, Germany

## Abstract

The protein family known as G-protein coupled receptors (GPCRs) comprises an important class of membrane-associated proteins, which remains a difficult family of proteins to characterize because their function requires a native-like lipid membrane environment. This paper focuses on applying a single step method leading to the formation of nanolipoprotein particles (NLPs) capable of solubilizing functional GPCRs for biophysical characterization. NLPs were used to demonstrate increased solubility for multiple GPCRs such as the Neurokinin 1 Receptor (NK1R), the Adrenergic Receptor â2 (ADRB2) and the Dopamine Receptor D1 (DRD1). All three GPCRs showed affinity for their specific ligands using a simple dot blot assay. The NK1R was characterized in greater detail to demonstrate correct folding of the ligand pocket with nanomolar specificity. Electron paramagnetic resonance (EPR) spectroscopy validated the correct folding of the NK1R binding pocket for Substance P (SP). Fluorescence correlation spectroscopy (FCS) was used to identify SP-bound NK1R-containing NLPs and measure their dissociation rate in an aqueous environment. The dissociation constant was found to be 83 nM and was consistent with dot blot assays. This study represents a unique combinational approach involving the single step de novo production of a functional GPCR combined with biophysical techniques to demonstrate receptor association with the NLPs and binding affinity to specific ligands. Such a combined approach provides a novel path forward to screen and characterize GPCRs for drug discovery as well as structural studies outside of the complex cellular environment.

## Introduction

G-protein coupled receptors (GPCRs) are a diverse family of membrane proteins that act as transducers of signals from extracellular ligands, e.g. photons, odorants, hormones, nucleotides, nucleosides, peptides, lipids and proteins, initiating a diverse range of intracellular responses. [1]–[3] They represent the most abundant drug target class of proteins. [4]–[5] Approximately 40–50% of all drugs used today modulate some form of GPCR activity. [6] Although over 350 human GPCRs have been previously described, their function is only speculative and there are many more putative orphan receptors (details regarding the receptor families can be found at http://www.iuphar-db.org). [7]–[8] Those GPCRs with unknown function are interesting candidates to identify as potential novel targets for future studies of their activity, which in turn may further lead to the discovery of new drugs. However, more efficient methods for production and characterization are needed.

Cell-free protein production has become a widely accepted means to speed up the production and characterization of this class of membrane proteins, as over-expression of membrane proteins *in vivo* typically results in cell toxicity, protein aggregation, misfolding, and low yield. [9]–[12] Cell-free expression can also alleviate problems such as the need for time-consuming cloning, cell transfection, cell growth, cell lysis, and challenges related to subsequent purification. [13] Cell-free systems permit unique labeling or tagging strategies not always readily available to whole cell systems for protein characterization. [13]–[14] They have also proven beneficial to structural studies by NMR and X-ray crystallography. [15]–[17] However, previously published studies of cell-free production of GPCRs typically required expression and subsequent purification combined with detergent solubilization of the proteins. [18] This often alters the conformation and function of these membrane proteins. To overcome these aforementioned problems, functional membrane proteins can be assembled in lipid/protein-based particulate structures connoted as “nanodiscs”, or nanolipoprotein particles (NLPs). [19]–[21] Such methods were previously used to express GPCRs and model proteins such as bacteriorhodopsin reconstituted into NLPs. [22]–[26] These nanoparticle complexes form a compelling approach for the stabilization and characterization of membrane proteins. [23], [25] Recent work has described the self-assembly of a single integral membrane protein into soluble nanoparticulate phospholipid bilayers. [22]–[23], [27]–[28] This approach has previously been applied to the adrenergic receptor β2, and rhodopsin reconstituted into NLP constructs [22]–[23], [29] and shown to efficiently activate the associated G protein (transducin for rhodopsin). This process, however, relied on the separate expression and detergent extraction of GPCRs of interest. [18]–[19].

We have previously demonstrated a single-step cell-free approach for the expression of nanodisc-associated bacteriorhodopsin (bR), [26], [30]–[31] a 7 transmembrane spanning protein, which is the structural model protein for rhodopsins and other GPCR family members. This complex was characterized using fluorescence correlation spectroscopy (FCS) and showed unique diffusion behaviour in solution. [26] Single molecule fluorescence techniques have been used to study GPCR interactions *in vivo* since the early 2000s [32]–[34] as well as to NLP associated complexes. [23], [35]–[36] These earlier studies set the stage for further development of FCS to address protein-protein associations combined with kinetic characterization of GPCRs.

In this paper we report the de novo synthesis of several active human GPCRs, and rapid solution-based functional binding studies using FCS, a single molecule fluorescence technique. Electron paramagnetic resonance (EPR) spectroscopy and fluorescent dot blot assays were used for comparison as well. Compared to the other assays, FCS provided a more quantitative approach to rapidly determine the solution-based binding constants for GPCR-ligand interactions. FCS was advantageous by requiring small volumes of material for kinetic assessment. Moreover, FCS can be extended to become a high-throughput cell-free screening platform for GPCRs.

## Results

**Figure 1:**
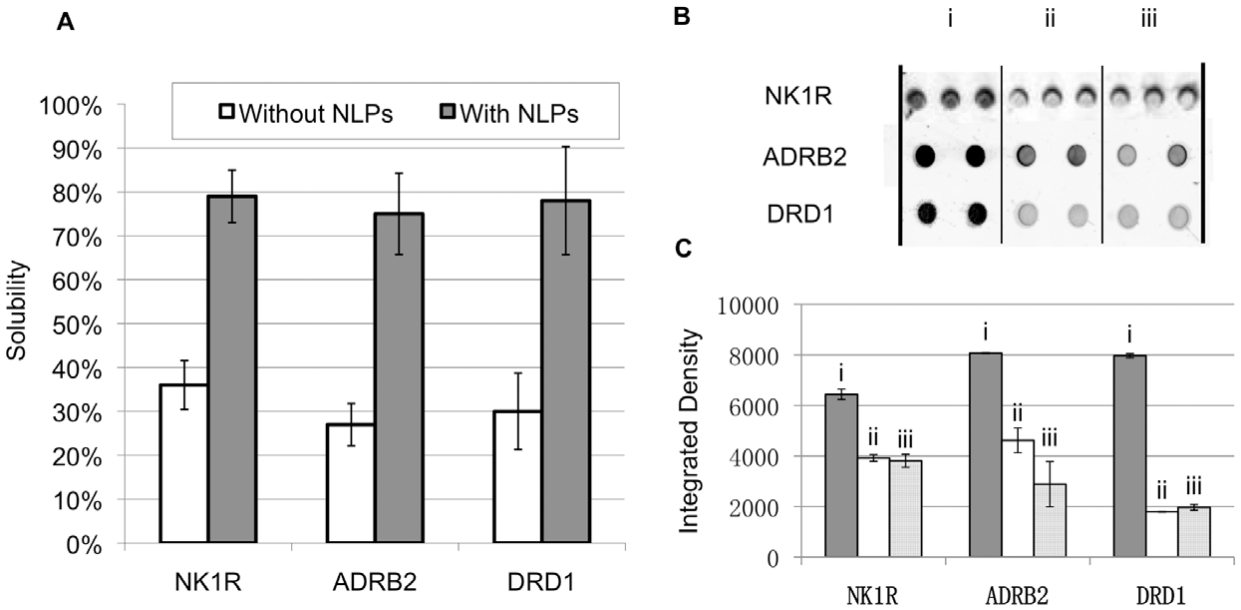
Co-expression with NLPs increased solubility for NK1R, ADRB2, and DRD1 and retained their activity. (a) Comparison of GPCR solubility when expressed with or without NLPs. NK1R, ADRB2, and DRD1 were expressed as GFP fusion proteins. (b) The dot blot of GPCR-NLPs when incubating with (i) 100 nM fluorescent-labeled ligand and (ii) 100 nM fluorescent-labeled ligand and 100 µM non-labeled ligand. (iii) Non-specific signal of 100 nM fluorescent-labeled ligand alone on the filter paper as the control. The tests were done with 3 replicates for NK1R, 2 replicates for ADRB2 and 2 replicates for DRD1. (c) Quantification of dot blot assay. The significant difference of fluorescence intensity between (i) and (ii), (iii) indicates activity of NK1R, ADRB2 and DRD1.

NK1R, ADRB2 and DRD1 were codon-optimized and co-expressed respectively with Δ49A1 in the presence of DMPC using a cell-free *E. coli* expression system. The NK1R protein was produced and purified to a total level of approximately 100 µg/mL. The material was estimated to have an NLP insertion rate of 17% ±5%. Figure 1a shows that the solubility increased significantly (more than 2 fold) for all 3 GPCRs when associated with NLPs as compared to when the GPCRs were expressed alone. Figure 1b shows dot blot assays using various fluorescently labeled ligands capable of recognizing NK1R, ADRB2 and DRD1. Figure 1c shows the quantification of specific ligand association for the dot blot assays. Compared with non-specific binding of fluorescent-labeled ligands (when excessive non-labeled ligands were added) to the GPCR-NLP complexes, the specific binding demonstrated much higher fluorescence intensity, indicating retained activity of all 3 GPCRs when co-expressed with NLPs.

**Figure 2:**
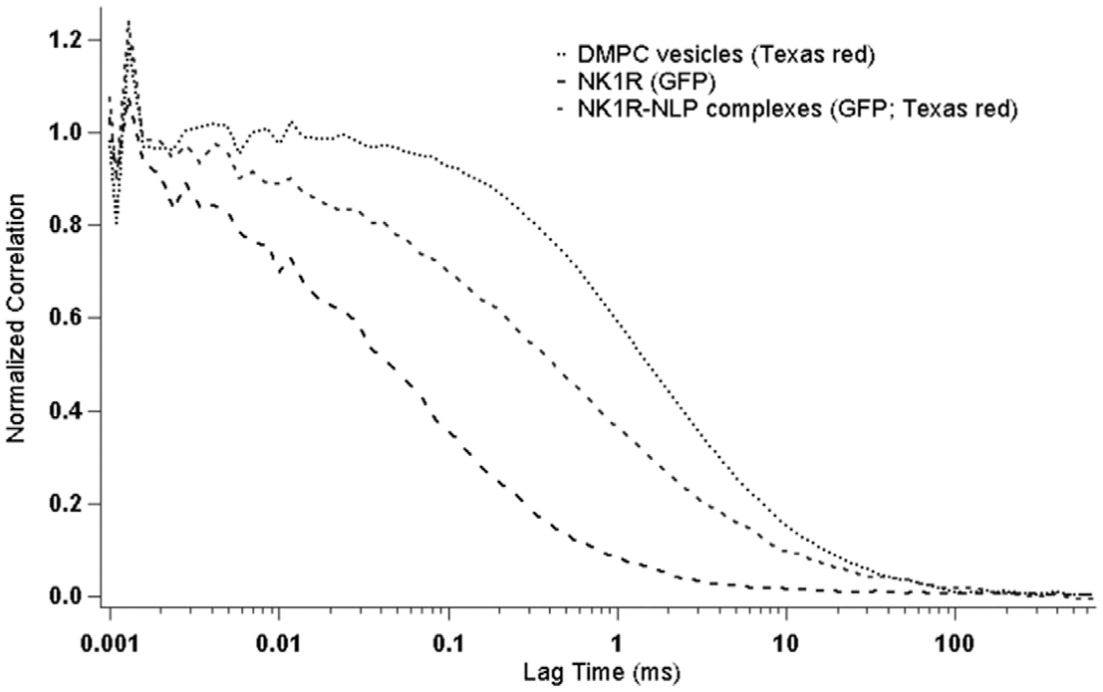
Diffusion curves of lipid vesicles, NK1R and NK1R-NLP complexes. The lipids and NK1R were labeled by Texas Red and GFP respectively. The cross correlation of Texas Red and GFP represents the interaction between lipids and NK1R, indicating the formation of NK1R-associated NLPs. The diffusion times of lipid vesicles, NK1R and NK1R- NLP complexes are 4.46, 0.17, and 0.51 ms respectively.

The formation of NK1R-NLPs was confirmed by FCS measurements of dual-labeled NK1R-NLP complexes freely diffusing in solution. Figure 2 shows the normalized diffusion curves of individual NK1R proteins, NK1R-NLP complexes, and lipid vesicles. NK1R can be distinguished by the green fluorescence of the GFP fusion that was constructed for this experiment. Lipid vesicles were identified by the red fluorescence of Texas Red-DHPE that was incorporated into the vesicles. For NK1R alone (hydrodynamic diameter: 4.9 nm, measured by particle sizer) we obtained a diffusion time of 0.17±0.025 ms, while the lipid vesicles yielded a diffusion time of 4.46±1.55 ms (hydrodynamic diameter on average: 73.0 nm, measured by particle sizer). To identify and isolate NK1R-containing NLP complexes, we determined the amount of cross correlation between GFP and Texas Red, the fluorophores on the protein and lipids, respectively. This positive cross correlation confirmed the formation of NK1R-loaded NLPs. Moreover their diffusion time of 0.51±0.37 ms indicates a diameter of 10.3 nm for these complexes.

**Figure 3:**
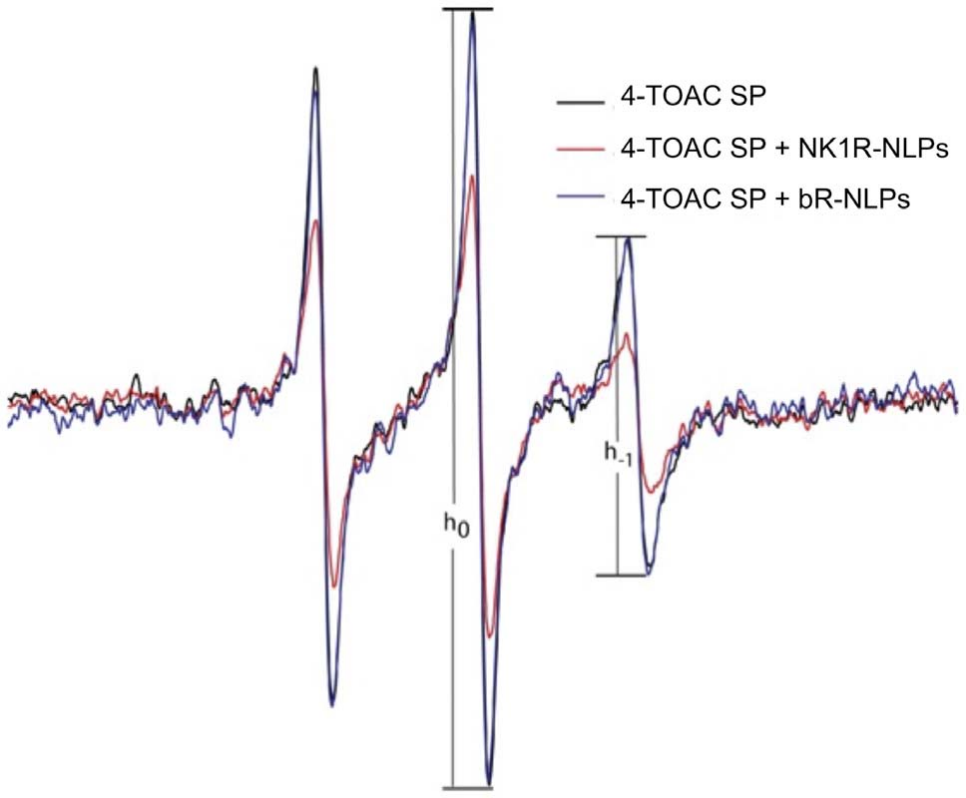
EPR spectra of 4-TOAC SP are sensitive to the presence of NLPs containing NK1R. 4-TOAC SP in buffer or combined with bR-containing NLP gave a narrower spectrum (h_−1_/h_−0_ ∼0.44). The spectrum for 4-TOAC SP was significantly broadened when NK1R-NLP complexes were present (red line, h_−1_/h_0_ = 0.34) reflecting the diminished rotational freedom of its receptor-bound state.

We have previously shown that a modified version of the Substance P peptide containing the TOAC spin-label at the position 4 (4-TOAC SP) binds and activates the NK1R protein in a native-like environment using the cell membranes containing the over-expressed receptor. [37] Furthermore, upon binding to the NK1R on the surface of mammalian cells, the change in rotational diffusion of the 4-TOAC SP can be detected by EPR spectroscopy. Since the SP binding pocket requires proper 3-dimensional folding of the receptor’s core helices, [37] we used 4-TOAC SP to evaluate the ligand binding properties of the NLP-solubilized receptor synthesized under cell-free conditions. Figure 3 shows the EPR spectrum of 4-TOAC SP in the presence of NLPs containing NK1R (red curve) compared to that in the presence of NLPs containing bR (blue curve) with respect to that in a buffer control (black curve). While the curves for the sample containing bR and buffer alone were identical, the sample containing NLP-solubilized NK1R showed a significantly broadened curve, indicating a substantial loss in rotational averaging. The increase in correlation time for the bound ligand resulted in inhomogeneous broadening, where the magnitude of change can be estimated by the peak-height ratio h_−1_/h_0_. [38] The relative peak-height ratio is taken as an empirical motional index for the spin label that was attached to SP. Typically a higher ratio represents a greater motion freedom of the attached spin label. In the absence of NK1R, 4-TOAC SP displayed a peak-height ratio of 0.43. The line shape of the 4-TOAC SP was similar to that in the presence bR-associated NLPs (with a h_−1_/h_0_ value of 0.44). However, in the presence of NLP-associated NK1R, the peak height ratio decreased to 0.34, indicating a substantial reduction on the rate of rotational diffusion experienced by 4-TOAC SP. This positive confirmation of binding between SP and NK1R-NLPs indicates that NK1R folds correctly in NLPs and retains its bioactivity.

**Figure 4:**
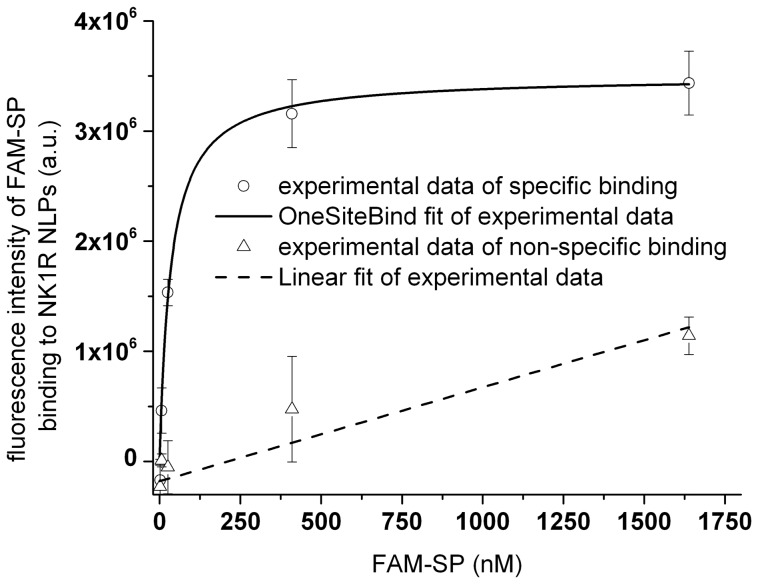
Saturation binding assay of FAM-SP on filter paper after interacting with NK1R-NLPs. The fluorescence intensity was averaged through 3 replicates with the error bar showing the standard deviation. Each data point represented intensity of different amounts of FAM-SP interacting with NK1R-NLPs subtracted by non-specific adsorption of the same amounts of FAM-SP to the paper. The solid curve represents specific binding. The dashed curve represents non-specific binding (NK1R-NLPs saturated by excessive amount of non-labeled SP). The binding curve was fit to an “OneSiteBind” model Y  =  B_max_ × X/(K_d_ + X), where Y represents fluorescence intensity caused by binding and X represents concentration of FAM-SP in the solution after reaction. The fitting results gave (3.5±0.3) ×10^6^ (fluorescence intensity) for B_max_ and 34±7.8 nM for K_d_ (dissociation constant).

To determine the binding affinity of FAM labeled SP (FAM-SP) interacting with NK1R-NLPs, reactions were tested using dot blot assays. We measured the fluorescence image of a dot blot containing 3 replicates of NK1R-NLPs binding with different concentrations of FAM-SP and another 3 replicates of NK1R-NLPs binding with the same series of FAM-SP but with excessive amounts of non-labeled SP included. For the control, there were additional 3 replicates of the ligand FAM-SP alone that were adsorbed on the filter paper using the same series of concentrations. After subtracting specific and non-specific signals by the control signal (FAM-SP only), the curve for specific binding was fitted to a OneSiteBind model where non-specific binding indicated a linear fit (Figure 4). This was consistent with our expectation that increasing amounts of FAM-SP bind to NK1R-NLPs until the reaction reaches saturation. Data analysis of the binding curve produced a dissociation constant of 34±7.8 nM.

**Figure 5:**
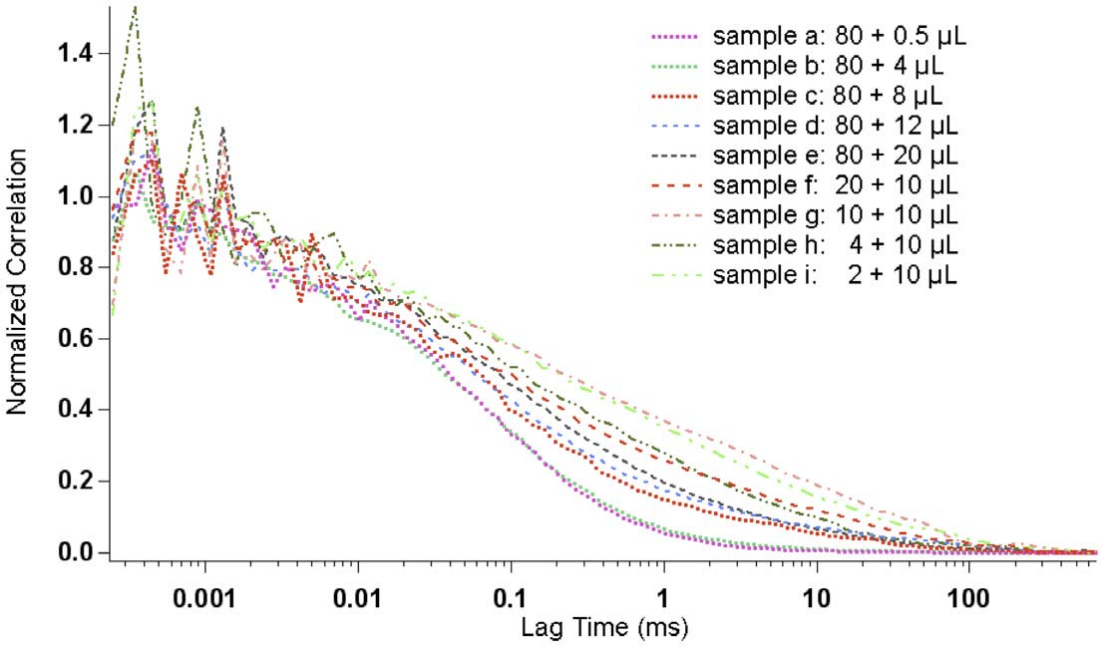
Diffusion curves of FAM-SP after binding with different amounts of NK1R-NLPs. Sample a to e represent 80 µL FAM-SP binding with 0.5, 4, 8, 12, 20 µL NK1R-NLP complexes respectively. Sample f to h represent 20, 10, 4, 2 µL FAM-SP binding with 10 µL NK1R-NLPs complexes respectively.

Compared with the dot blot assay, FCS is a more accurate method to rapidly study the binding affinity of FAM-SP interacting with NK1R-NLPs in aqueous solution without substrate and thus exhibiting significantly reduced non-specific binding. Here, we measured the diffusion time of FAM-labeled SP by FCS after adding different concentrations of NK1R-NLPs. Since the FAM-labeled SP has a significantly smaller hydrodynamic radius than the NLPs, we expected a significant increase in diffusion time upon interaction of SP with NK1R-NLPs. Figure 5 shows that after increasing the total number of NK1R-NLPs by adding larger volumes of NK1R-NLP suspension, the diffusion curves for FAM-SP continuously shifted to longer diffusion times. This indicates that a larger number of FAM-SP were interacting with NK1R-NLPs forming bigger-size species in solution, and therefore caused an increase in diffusion times, until the binding of FAM-SP to NK1R became saturated.

**Figure 6:**
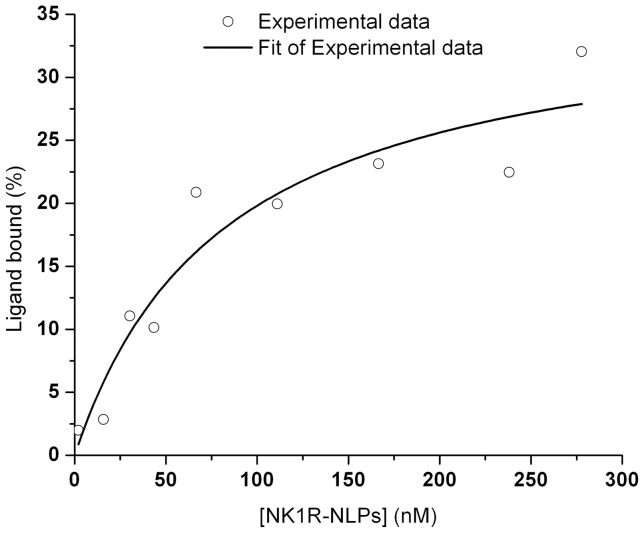
Saturation binding curve of NK1R-NLPs to FAM-SP. [NK1R-NLPs] is the concentration of free NK1R-NLPs at equilibrium and was calculated from the subtraction of the amount of NK1R-NLPs bound with FAM-SP from the total amount of NK1R-NLPs added. The ligand bound % was calculated by comparing the average diffusion time of the mixture (free FAM-SP and FAM-SP bound with NK1R-NLPs) with the individual controls (diffusion times of free FAM-SP and free NK1R-NLPs). The experimental data (blue dots) were fitted to an “OneSiteBind” model Y  =  B_max_ × X/(K_d_ + X). Y represents the percentage of bound ligand in the total amount of ligand, and X represents the concentration of NK1R-NLPs in the solution after reaction. The fitting results in 36±5.6 nM for B_max_ and 83±33 nM for K_d_.

Each of the individual diffusion curves for FAM-SP were fitted to a 2-species complex diffusion model. [26] The average diffusion times were determined as a result of the fit parameters for bound FAM-SP and unbound FAM-SP. Since we also had control data of diffusion times for FAM-SP and NK1R-NLPs alone, by comparing the average diffusion times of the mixture with the controls, we were able to infer the percentage of bound FAM-SP (Ligand bound %) versus the total amount of FAM-SP added and the concentration of free NK1R-NLPs at equilibrium ([NK1R-NLPs]). By plotting Ligand bound % versus [NK1R-NLPs] and fitting it to an “OneSiteBind” model (Figure 6), we calculated the dissociation constants K_d_ and B_max_. The results are 83±33 nM and 36±5.6 nM, respectively. This dissociation constant is consistent with the dot blot assays (K_d_  = 34±7.8 nM) in the range of tens of nanomolar but albeit 2∼3 fold higher when measured by FCS. FCS measured ligand binding directly in solution and maintained an aqueous environment where background and non-specific binding were minimal. Compared with results from dot blot assays, the fitting results obtained from FCS are considered more accurate when the background/noise and non-specific binding were seen in the dot blot assays but not included for the fitting analysis.

## Discussion

Compared to other approaches for obtaining membrane-bound receptor proteins, cell-free co-expression provides a one-step viable method to produce functional GPCRs such as NK1R. The NLP serves as an ideal membrane mimetic that renders the protein soluble and thus easily accessible for ligand binding studies using methods such as EPR spectroscopy and FCS. Compared to other “nanodisc” or NLP based studies, we showed the first functional GPCR through de novo expression using the DNA sequence representing the full-length protein, independent of a fusion protein for stabilizing the receptor. Furthermore, we were able to demonstrate kinetic characterization of the solubilized receptor using FCS. For comparison, in a recent publication describing the cell-free synthesis of functional adrenergic receptor β2 complexed with nanodiscs, [39] the receptor required insertion of a T4 lysozyme sequence in the loop region to obtain functional adrenergic receptor β2 protein. Using our method NK1R, ADRB2 and DRD1 were all functional in ligand binding assays after a single-step co-expression and co-assembly system without requiring detergents or protein modification for stabilization. It is also worth noting that in other nanodisc-related GPCR studies or cell-free production of GPCR assays, separate protein production and purification preprocessing with detergents was required prior to NLP complex assembly. [29] Our results indicate that adding additional purification steps can be avoided as well as the requirement for using a fusion protein for stabilizing the GPCRs.

Assessment of NK1R activity was independently validated by three different methods that included fluorescent dot blot assays, EPR spectroscopy and FCS. Dot blot assays and EPR spectroscopy demonstrated that NK1R loaded into NLPs were bioactive. Furthermore, the nM affinities were comparable to earlier published studies using mammalian derived NK1R. [37] Among these three approaches, FCS is a particularly powerful tool for characterizing NLPs, as it provided a more quantitative approach to rapidly determine the solution-based binding constants for NK1R-SP interaction studies. FCS also enabled us to determine the hydrodynamic radii of the diffusing complexes along with their concentrations (based on the amplitude of the correlation function). In addition, FCS was advantageous by requiring less material (proteins) in volumes as small as ∼10 µL for kinetic assessment in our studies. The measurments are typically rapid and take ∼5 minutes. However, as it requires concentrations of ∼100 nM or less of fluorescently labeled compounds, the main challenge of FCS is its limited dynamic range for interaction analysis. This can be overcome by an appropriate design of a combinatorial screen of initial concentrations for NK1R-NLPs and SP. Mixing fluorescently labeled compounds with appropriate amounts of unlabeled compounds is the strategy for extending the concentration range. After reaching equilibrium, the actual concentrations of each species were then inferred and used to calculate the dissociation constant.

The technique of FCS can be generalized for screening multiple GPCRs to assess binding constants as well as drug binding studies. The most popular method for screening binding activity for GPCRs is using radioactivity assays, however this is often disadvantageous since it requires the handling of isotope labeled ligands. Other screening approaches include dot blot assays and EPR spectroscopy as described above. All of these methods require larger amounts of reagents that are not always easily achievable for the GPCRs of interest. In comparison, FCS can be performed in small volumes (∼10 µL) or even less when microfluidic delivery methods are employed, and it is very sensitive to concentrations as in the range as low as picomolar. Therefore, generalizing the method of using FCS to assess binding constants for screening other GPCRs is warranted.

Lastly, FCS can be extended to become a high-throughput cell-free screening platform for GPCRs by facilitating simultaneous measurements in multi-well plates, providing real-time monitoring of production, purification, and functionality of GPCRs as well as other synthetic receptors based on the cross correlation between signals from the proteins and their specific ligands as demonstrated here. Furthermore, the diffusion curves provide detailed structural information about the particular association between GPCRs and NLPs to form complexes as well as monitoring interactions between GPCRs and specific ligands or other small molecules such as lipids.

In contrast to cell-based assays, our approach is currently limited to demonstrating ligand binding for a very specific set of GPCRs. Although ligand binding alone does not conclusively demonstrate the entire protein is folded natively, it does show that critical tertiary structure is achieved since the binding of tachykinins has been shown to involve liganding from residues on a least 3 different transmembrane domains. [40] GPCRs involve a greater level of complexity. This includes G-protein activation and receptor internalization, which are more complete measurements of GPCR function. [5] Because our system also lacks post-translational modifications, cell membrane lipid components, and the heterotrimeric G-protein, we do not expect our assays to fully mirror the protein in a cell membrane. In the future, such studies could be possible using techniques such as FCS within cells. FCS is highly amenable to measurements in solution utilizing cross-correlating measurements, which would potentially allow measurements in heterogeneous environments such as cell membranes and cell fractions. [32]–[33] In the future such experiments could be designed to better access both the *in vitro* and *in vivo* biology of GPCRs complexed with NLPs.

In summary, we applied a de novo synthesis, cell-free co-expression, and *in-situ* analysis method to produce nanolipoprotein particles (NLPs) capable of solubilizing three GPCRs (NK1R, ADRB2 and DRD1) while maintaining their biological activity. We also demonstrated a robust method for assessing binding constants for NK1R-NLPs that interact with SP using FCS. This combined approach should be capable of high-throughput screening for active GPCRs produced by cell-free co-expression. In the future, it will be of interest to build upon these studies to explore mechanisms behind G-protein activation and potential receptor uptake in cells.

## Materials and Methods

### Materials

FAM (Fluorescein amidite) labeled Substance P was purchased from Anaspec Inc. (Fremont, CA). DMPC (1, 2-ditetradecanoyl-sn-glycero-3-phosphocholine) was purchased from Avanti Polar Lipids, Inc. Texas Red® DHPE (Texas Red® 1, 2-dihexadecanoyl-sn-glycero-3-phosphoethanolamine triethylammonium salt), the SDS and native PAGE kits were purchased from Life Technologies. Δ49A1 (a truncated apolipoprotein A-1) and bOp sequence, which encodes bR, were described previously. [30] The Δ49A1 sequence was cloned into pIVEX2.4b vector. Protein sequences for NK1R, ADRB2 and DRD1 were obtained from the National Center for Biotechnology Information (NCBI) protein database. The corresponding DNA sequences were codon optimized for *E. coli* expression by DNA2.0 and cloned into a pJexpress414 vector. Information about all the sequences is shown in Table S1. RTS 500 ProteoMaster Kits were purchased from Roche Molecular Diagnostics. The fluorescent-labeled antagonists for ADRB2 and DRD1 were purchased from CellAura Technologies.

### Lipid Preparation

Small unilamellar vesicles of DMPC were prepared by probe sonicating a 25 mg/mL aqueous solution of DMPC on ice until optical clarity was achieved, typically for 15 minutes. Two minutes of centrifugation at 13,700 RCF was used to remove any metal contamination from the sonication probe tip. DMPC small unilamellar vesicles were added to the cell-free reaction at a final concentration of 2 mg/mL.

### Expression, Purification and Solubility Tests of GPCR-NLP Complexes

A myriad of commercial cell-free kits, to include mammalian, yeast, insect, *E. coli* and others, are available for providing cell-free expressed proteins. [31], [39] For this study preparative 1 mL reactions were carried out using the Roche RTS 500 ProteoMaster Kit to optimize for protein yield combined with functional ligand binding. Lyophilized reaction components (Lysate, Reaction Mix, Amino Acid Mix, and Methionine) were dissolved in reconstitution buffer and combined as specified by the manufacturer. DMPC was used either as non-labeled or as fluorescently labeled by mixing 99.5% DMPC and 0.5% Texas Red® DHPE (Molar concentration). To co-express GPCRs and Δ49A1, different ratios of plasmids were added to the lysate mixture along with added DMPC vesicles for screening. The reactions were incubated at 30°C overnight. SDS-PAGE was used to confirm the protein yields [30] and determine the optimized plasmid ratios between the Δ49A1 plasmid and GPCR expression vector. The optimized ratios 100∶1 for NK1R: Δ49A1, 20∶1 for ADRB2: Δ49A1 and 20∶1 for DRD1: Δ49A1 were used to produce GPCR-NLP complexes for characterization. To characterize the solubilities of GPCRs produced by cell-free co-expression method, the relative fluorescence units (RFU) were measured for the supernatant solution after spinning the vials for 10 minutes at an rpm of 13K and then taking the spectrum using a Nanodrop ND-3300. [13].

Immobilized metal affinity chromatography was used to isolate the protein complex of interest (Δ49A1 or Δ49A1 associated with GPCR of interest) using the Δ49A1 encoded His tag. None of the GPCR proteins contained a His tag. The soluble fraction (∼1 mL) was mixed with Ni-NTA Superflow resin (1 ml of 50% slurry, Qiagen) according to the manufacturer’s protocol using native purification conditions with the following modifications: 12-column volumes of 10 mM imidazole in PBS buffer was used to wash the column. A total of 6 mL of elution buffer (400 mM imidazole in PBS) were used to elute the bound protein in 1-mL aliquots. All of the elution fractions were combined, concentrated and buffer exchanged into TBS using a 100 kDa molecular weight sieve filter (Vivascience) to achieve a final volume of ∼200 µL. This material was used for all subsequent characterization assays. The purification quality was confirmed by SDS PAGE. [30].

### Dot Blot Assays of Fluorescence Labeled Ligands Binding with GPCR-NLP Complexes

A total of 100 µL of 100 nM fluorescence labeled ligands for NK1R, ADRB2 and DRD1 were mixed with 100 µL ∼0.2 nM GPCR-NLPs. The reactions were allowed to mix in binding buffer (50 mM Tris-HCl pH 7.4, 3 mM MgCl_2_ containing protease inhibitor with 0.04 mg/ml BSA and 100 mM NaCl) for 1 hour at room temperature. Several replicates (3 replicates for NK1R and 2 replicates for ADRB2 and DRD1) were then blotted on the filter paper. The same procedure was used to determine non-specific binding when 100 µL of the 10 µM non-labeled ligands were also included. For the control, 100 µL of 100 nM fluorescence labeled ligands alone were added onto the filter paper. Then the filter paper was rinsed 5 times with 100 µL ice-cold rinsing buffer (25 mM Tris pH 7.5, 3 mM MgCl_2_, 1 mM EDTA) each time and dried in a 37°C incubator. Signals were measured using a fluorescence imaging system GE Typhoon 9410. The NK1R lignad (FAM-SP) was excited at 488 nm and signals were collected using a 520/40 nm bandpass filter. Fluorescent-labeled ligands for ADRB2 and DRD1 were excited at 633 nm and signals were collected using a 670/30 nm bandpass filter.

To obtain the binding curve of NK1R interacting with FAM-SP, the same procedure was used for FAM-SP at a series of concentrations (0.1, 0.4, 1.6, 6.4, 25.6, 102.4, and 1638.4 nM). To test non-specific binding, 100 µL of 10 µM non-labeled SP was included with FAM-SP. For the control, FAM-SP alone at the same series of concentrations was added onto the filter paper. The experiment was run in replicate 3 times.

### FCS Characterization of NK1R-NLP Complexes and Binding Assay of FAM Labeled SP Interacting with NK1R-NLP Complexes

Lipid vesicles formed by DMPC were labeled by addition of a small fraction of fluorescently labeled DHPE (Texas Red dye 0.5% volume percentage). NK1R was labeled with a GFP fusion built into its plasmid during translation. In order to confirm the formation of NK1R-NLPs, the diffusion times of fluorescently labeled species in a volume of 10 µL were measured by FCS (MicroTime200, PicoQuant, Berlin, Germany). The samples were excited by a 470 nm laser (Picoquant pulsed diode laser, 70 ps pulse width, 20 MHz repetition rate) and the time traces of fluorescent signals were collected for 10 minutes. The focus of the confocal FCS system was adjusted to be 5 µm above the cover slip surface before each measurement, so that the FCS measurements were performed at a defined distance above the glass surface. The pinhole size of the fluorescence detection system was set to 100 µm in diameter. After the pinhole adjustment, the fluorescence light was divided via a 50/50 beam splitter cube, passed an emission filter and focused onto 2 SPCM-AQR SPAD detectors (Perkin Elmer). All measurements were performed using the SymPhoTime Software (PicoQuant) and the diffusion curves were plotted using Igor Pro 6.05A.

For the binding assay, FAM-SP was diluted to a concentration of 1 µM in binding buffer (50 mM Tris-HCl, 3 nM MgCl_2_, 100 mM NaCl, 0.04 mg/mL BSA, pH 7.5). 80 µL NK1R-NLPs (non-labeled and without fusion GFP, ∼ 1 mg/mL in protein concentration) were mixed with 0.5, 4, 8, 12, and 200 µL 1 µM FAM-SP respectively, and then incubated for 1 hour at room temperature (∼25°C). 10 µL 1 µM FAM-SP was mixed with 20 and 10 µL NK1R-NLPs, respectively, and then incubated for 1 hour at room temperature. After incubation, 10 µL of each individual mixture was assayed using FCS. The data were collected using SymPhoTime Software. The diffusion curves were plotted using Igor Pro 6.05A and analyzed using OriginPro 8.

### EPR Spectroscopy Measurements of Spin Labeled SP Binding to NK1R-NLP Complexes

EPR measurements were carried out in a JEOL TE-100 X-band spectrometer fitted with a loop-gap resonator that was used for EPR spectroscopy measurements as described previously. [37] SP with TOAC spin label was added to either buffer or NLP preparation to a final concentration of 10 µM and immediately loaded (∼5 µl) into a sealed quartz capillary tube and measured by EPR spectroscopy. The spectra were obtained by averaging signals from three 2-minute runs, with a sweep width of 100G. 4 mW microwave power was used and modulation amplitude was optimized to the natural line width of the attached spin probe. All the spectra were recorded at room temperature.

## Supporting Information

Table S1 **Genes and vectors used for protein expression.**
(DOC)
