# Supplementary material for: Characterization of De Novo Synthesized GPCRs Supported in Nanolipoprotein Discs
Source: PLoS One. 2012 Sep 28;7(9):e44911. doi: 10.1371/journal.pone.0044911 (PMC3460959; doi:10.1371/journal.pone.0044911)
Supplement: Table S1 — Genes and vectors used for protein expression. (DOC) [file pone.0044911.s001.doc]

**Table S1**. **Genes and Vectors used for protein expression.**

| **Expression plasmids** | | | |
| --- | --- | --- | --- |
| Encoded gene | Vector | Tag | Codon optimized for *E. coli*. expression |
| NK1R | pJexpress 414 | None | Yes |
| NK1R-GFP | pJexpress 414 | GFP | Yes |
| ADRB2 | pJexpress 414 | None | Yes |
| DRD1 | pJexpress 414 | None | Yes |
| Δ49ApoA1 | pIVEX2.4b | 6xHis | No |
